# Benefits of the microalgae *Spirulina* and *Schizochytrium* in fish nutrition: a meta-analysis

**DOI:** 10.1038/s41598-023-29183-x

**Published:** 2023-02-07

**Authors:** S. Trevi, T. Uren Webster, S. Consuegra, C. Garcia de Leaniz

**Affiliations:** Swansea University, Centre for Sustainable Aquatic Research (CSAR), Singleton Park, Swansea, SA2 8PP UK

**Keywords:** Zoology, Environmental sciences

## Abstract

Use of microalgae in fish nutrition can relieve pressure on wild fish stocks, but there is no systematic quantitative evaluation of microalgae benefits. We conducted a metanalysis on the nutritional benefits of *Spirulina* and *Schizochytrium* as replacements of fishmeal and fish or plant oil, respectively. We reviewed 50 peer-reviewed studies involving 26 finfish species and 144 control vs microalgae replacement comparisons. Inclusion of *Spirulina* in the fish diet significantly improved growth compared to controls (SMD = 1.21; 95% CI 0.71–1.70), while inclusion of *Schizochytrium* maintained the content of omega-3 PUFA of the fish fillet compared to fish fed on fish or plant oils (SMD = 0.62; 95% CI − 0.51–1.76). Benefits were apparent at replacement levels as low as 0.025% in the case of *Spirulina* and 10% in the case of *Schizochytrium* oil. Dose-dependent effects were found for *Spirulina* replacement on growth, but not for *Schizochytrium* on omega-3 fillet content. Subgroup analysis and meta-regression revealed that ~ 24–27% of variation in effect sizes can be accounted by variation between fish families, the rest likely reflecting variation in experimental conditions. Overall, the evidence indicates that *Spirulina* and *Schizochytrium* replacement in aquafeeds can be used to improve fish growth and maintain fillet quality, respectively, but considerable uncertainty exists on the predicted responses. To reduce uncertainty and facilitate the transition towards more sustainable aquafeeds, we recommend that feeding trials using microalgae are conducted under commercially relevant conditions and that greater care is taken to report full results to account for sources of heterogeneity.

## Introduction

Global demand for fish products is expected to reach 186 M Tn by 2030 mostly driven by aquafeeds used in fish farming^1^. Aquafeeds represent the main cost in fish farming, and are also the area where sustainability can improve the most^2^. The main source of protein and lipids in aquafeeds has traditionally been marine groundfish and small pelagics, as they provide a good balance of the essential amino acids and the omega-3 fatty acids needed by virtually every commercially farmed fish^3^, and the high quality fish fillets needed for human consumption^4^. However, groundfish and small pelagics have declined worldwide as a consequence of the increasing demands made by aquaculture industry^5^.

In response to a shortage of wild fish, the aquafeed industry turned to plant-based ingredients due to their wider availability, lower costs and established knowledge from their use in human and livestock nutrition^6,7^. Plant oils from soyabean, linseed, flaxseeds, canola, palm and coconut became the prime candidates to replace marine oils, but their use in aquafeeds has several nutritional limitations as well as their own sustainability issues^8^. Livestock across the globe already rely heavily on plant oils, and there are fears that further demand from aquaculture could increase prices and lead to farmland expansion, putting more pressure on natural habitats^9^. Proteins derived from plants typically lack some of the essential amino acids present in fish meal, and some contain anti-nutritional factors, which can induce inflammatory effects with adverse effects on health, welfare and productivity^10^, while plant oils are typically deficient in n-3 LC-PUFA (omega-3) fatty acids^11^. These limitations are particularly problematic for marine farmed fish, as they cannot synthesize omega-3 fatty acids efficiently and must rely on the diet to obtain them^12^.

In a quest to find more sustainable alternatives to fish products, and more suitable sources of omega-3 fatty acids than plant oils, photosynthetic microalgae and cyanobacteria have received increasing attention^13^. The protein content and fatty acid composition of some microalgae are similar to those provided by marine pelagic fish, and are more nutritious and healthier for human consumption those derived from terrestrial plants^14^. Recent developments in algal biotechnology have also made the production of microalgae cheaper and more readily available^15^, but there are still challenges concerning upscaling, and knowledge gaps that have prevented their wider use. Early research on microalgae as aquafeeds focused on their use as feed additives, mostly as live cells, but there is increasing interest in their potential value as full or partial replacements of fish oil^16^ or protein^17^. Recent research has tended to focus on microalgae extracts as they are typically more digestible and less likely to include anti-nutrients than whole algae^16,18,19^, while large scale production of purified microalgae oils for incorporation into aquafeeds has become more efficient^20^.

One microalgae in particular, the genus *Arthrospira* (*Spirulina*), has received much attention as it has a protein content similar to that of marine fish^22^ as well as a high digestibility due to the lack of a cellulose cell wall^23,24^. With a global annual production of 3,000 Tn dry weight, the *Spirulina* market was worth $394 million in 2019, and is growing at a rate of ~ 10% annually. It is one of the most intensively farmed microalgae in aquaculture and the species that offers some of the best options for fish protein replacement^25^. However, *Spirulina* cannot be used as replacement for fish oil, as this requires microalgae with different nutritional profiles. The genus *Schizochytrium* is rich in omega 3 fatty acids, especially DHA^26,27^, and is already produced on an industrial scale as a food supplement^28^. It can also be incorporated into aquafeeds to improve the DHA content of the fish fillet^16^ and can be produced in the large quantities required by the salmon farming industry^29^.

A combination of *Spirulina* and *Schizochytrium* could be used as a replacement of fish protein and fish oil in aquafeeds^27^, but there is little guidance on optimal levels of replacement, and uncertainty regarding the extent to which the benefits of using microalgae can be generalised across different fish species. Production costs of live microalgae for aquafeeds currently range between 300 and 600 €/kg, and although these could be reduced by 60–80% with upscaling^30^, they are still more expensive than animal feedstuffs^16^. Crucially, there is no information regarding variation in effect sizes (i.e. variation in the magnitude of any purported microalgae nutritional benefits) and therefore it is not possible to assess to what extent the high costs of producing microalgae can be compensated by improved growth or enhanced fillet quality.

To address these questions, we carried a systematic review followed by a metanalysis on the effects of using *Spirulina* and *Schizochytrium* as replacement of fish protein and fish oil in fish feeds. Our aims were three: (1) to assess the extent of variation in the nutritional benefits of two of the main microalgae used in aquafeeds, (2) to gain insights into sources of heterogeneity and (3) to assess the existence of publication bias against negative results as this might have exaggerated the nutritional benefits of microalgae-enriched diets.

## Methods

### Selection criteria for the systematic review

**Figure 1:**
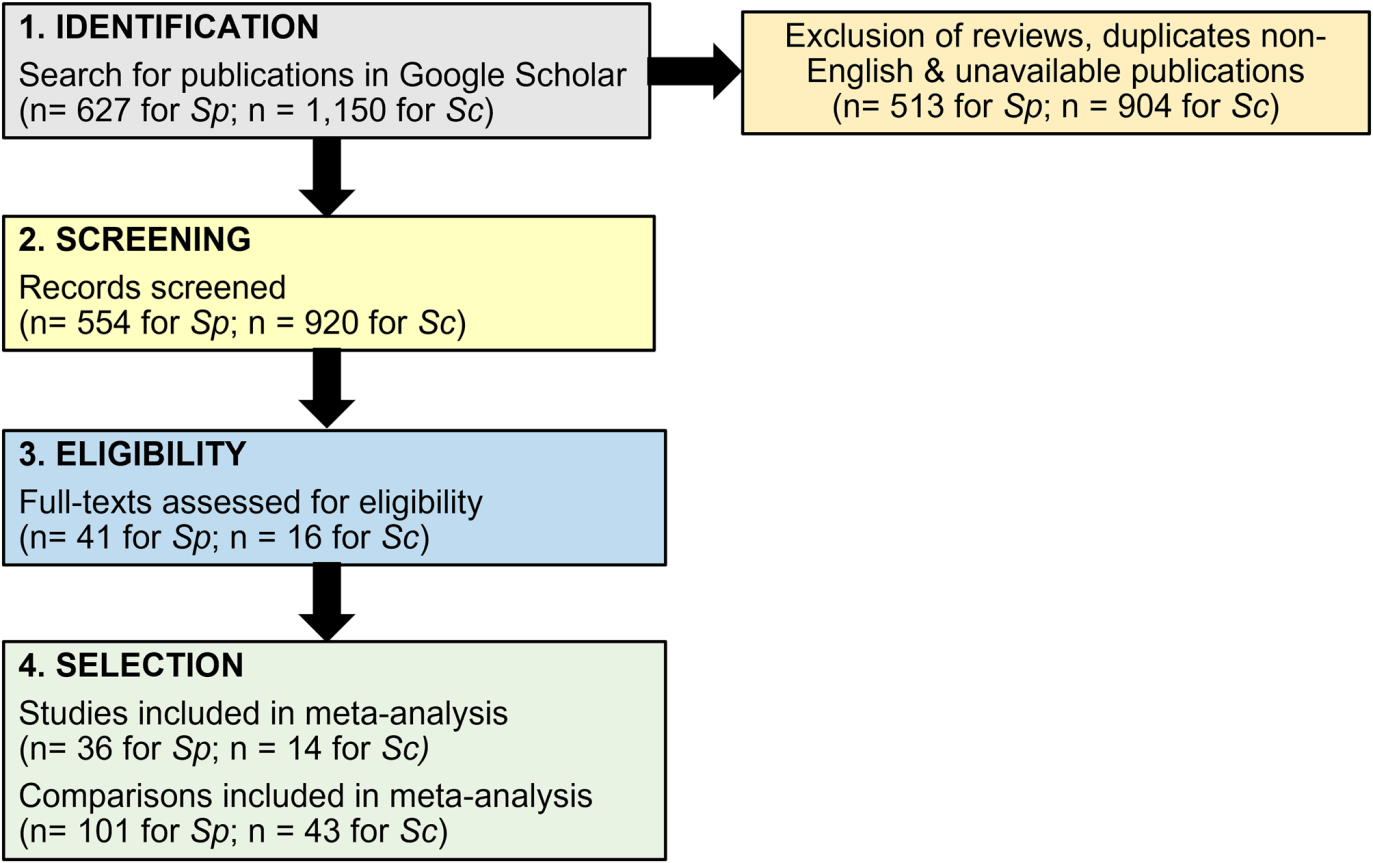
PRISMA workflow used to select publications for inclusion in the meta- analysis of nutritional benefits of microalgae in fish. *Sp* *Spirulina* dataset, *Sc* *Schizochytrium* dataset.

We adopted the PRISMA protocol (Preferred Reporting Items for Systematic Reviews and Meta-Analyses) as described by Moher et al.^31^ for the systematic literature review (Fig. 1). We searched Google Scholar with the keywords “*Spirulina*” AND “SGR” (Specific Growth Rate) AND “fish” AND “aquaculture” AND “Arthrospira” for the *Spirulina* analysis. This search string returned 627 results. For the *Schizochytrium* analysis, we searched for the keywords “*Schizochytrium”* AND “omega-3” AND “fish” AND “aquaculture”, obtaining 1150 results. The searches were carried out on 08/11/2019 and the timeline was set between the years 2000 and 2019 (inclusive), as before 2000 microalgae were used mainly as whole feed rather than as replacements in aquafeeds.

We used three criteria for selecting articles for subsequent analysis: (1) primary peer-reviewed research papers (i.e. we excluded reviews) carried out on finfish and written in English, (2) studies in which microalgae were used as partial replacement in fish feeds, and not as sole nutrients, and (3) studies that reported the Specific Growth Rate (SGR) for *Spirulina*, or the omega-3 content in the fish fillet for *Schizochytrium,* along with standard errors (or standard deviations) and sample size.

### Data extraction

The following data were obtained from the selected papers: (1) first author and year of publication, (2) mean value of the specific growth rate (SGR, for the *Spirulina* dataset) or omega-3 content of the fillet (for the *Schizochytrium* dataset) for the treatment (M*e*) and control groups (M*c*), (3) standard deviations of M*e* and M*c*, denoted as S*e* or S*c*, (4) number of fish sampled from the treatment (N*e*) and control (N*c*) groups; when fish were sampled as a batch each batch counted as one sample only, (5) scientific name and family, (6) habitat (freshwater—FW; saltwater—SW), (7) diet (carnivorous, C; omnivorous, O; herbivorous, H), (8) initial mass of the fish (g), (9) replacement level, expressed as %, of fish meal or fish oil and plant oil replaced with microalgae, (10) duration of the trial (days), (11) level of replication (number of tanks); (12) fish density (No. fish per tank) and (13) type of data (data obtained from individual fish, or pooled from batch measurements).

### Data analysis

We used R v3.5.1.^32^ for all statistical analysis. Study effect sizes were calculated as standardized mean differences (SMD) between the micro-algae enriched diet and the control diet (without micro-algae) adjusted for small sample size via Hedges’ *g* correction^33^. After inspection of the data, a random effects model was chosen to derive the overall effect^34^, since a single underlying common effect (fixed effect model) could not be assumed. Although a random effects model has wider confidence intervals than a model that assumes a common fixed effect, it is more realistic and also enabled us to examine how effect sizes varied across populations^35^. To fit the random model, we used the in between-study-variance HKSJ estimator method^36^ in the *meta* and *metafor* R packages. Forest plots were used to visualize the outputs of the meta-analysis.

We examined three measures of heterogeneity among studies: Cohran’s Q, with a cut-off of *P* = 0.10^37^, the *I*^2^ index which varies from < 25% to > 75% for small and substantial levels of heterogeneity, respectively^37^, and tau-squared (*τ*^*2*^), which represents the between-study variance^38^.

Evidence for publication bias was assessed by inspection of funnel plots^39^, followed by Egger’s linear regression test of funnel plot asymmetry^40^ and by the *P*-curve method^41^. Funnel plots compare the observed distribution of effect sizes on the x-axis against their standard error on the y-axis, which is typically inverted. In the absence of publication bias, studies should be contained within a symmetrical funnel at both sides of the pooled effect size. Studies that lie outside the funnel might indicate the existence of publication bias, although high heterogeneity can also result in asymmetrical funnels^41^. The *P*-curve method compares the significance level of the significant effect sizes against a theoretical left skewed and flat distributions, on the assumption that the most significant results should also be the rarest^42,43^. It can be used as a diagnostic tool for assessing the presence of publication bias, although it is also affected by high study heterogeneity^41^, and is most useful when heterogeneity is small to moderate (i.e. *I*^2^ < 50%).

Dose-dependent effects (i.e. to what extent the nutritional benefits of micro-algae depended on replacement levels) were assessed via mixed-effects meta-regression with the Sidik-Jonkman estimator for *τ*^*2*^ in the *dmetar* R package, using replacement, fish size, family, habitat, and feeding guild as predictors. Inspection of AIC values was used to arrive at the minimal adequate model.

### Outlier detection

We employed two methods to detect potential outliers and overly influential studies using the *dmetar* R package^41^: the ‘*find.outliers*’ function using a random effects model and “baujat” plots to help identify studies with a large overall contribution to the overall heterogeneity and a large influence on the pooled results^44^. Models were refitted after exclusion of outliers and overly influential points.

### Subgroup analysis

To gain insights into potential sources of variation in the benefits of using microalgae we carried out a subgroup analysis according to fish family, habitat (freshwater or marine), broad feeding guild (carnivore, omnivore, herbivore) and type of measurements (data collected from individual fish or pooled from a batch).

### Ethics statement

Our study did not involve any new experimental work on living animals, it compiled and analysed data already in the public domain. The study was approved by Swansea University College of Science Ethics Committee with number STAFF_BIOL_2119_160123150855_3 and complies with the ARRIVE guidelines for reporting research involving animals (https://arriveguidelines.org).

## Results

### Effects of *Spirulina* replacement on specific growth rate (SGR)

We found 36 quantitative studies on the effects of *Spirulina* replacement on fish growth (representing *k* = 101 control-treatment comparisons) that met the selection criteria and that were published during the period 2000–2019. These were carried out in 17 species belonging to 11 different fish families, mostly juveniles (weight range = 0.02–131 g) living in freshwater (88%), and having a herbivore or omnivore diet, including tilapia (*Oreochromis* sp.—37% of studies), various cyprinids (10% of studies) and catfishes (8% of studies). In most cases (83%), studies were carried out in triplicate tanks and involved an average of 34 individuals per tank (SD = 62), with feeding trials typically lasting between 70 and 120 days (Table 1). Replacement levels of *Spirulina* varied from 0.025 to 45% (mean = 8.9%, SD = 9.9).

**Table 1:** Results of feeding studies assessing the effects of *Spirulina* replacement on Specific Growth Rate (SGR, %) of farmed fish.

Author	Study ID	SMD	Me	Se	Mc	Sc	Ne	Nc	Species	Family	Hab	Diet	Size (g)	Replac. %	Days	Tanks	Dens	Data
El-Sheekh (2014)	sp1	0.3711	4.69	0.27	4.59	0.27	30	30	*O. niloticus x O. mossambicus*	Cichlidae	FW	H	0.206	14.00	65	3	10	Indiv
El-Sheekh (2014)	sp1	3.6974	5.30	0.03	4.59	0.27	30	30	*O. niloticus x O. mossambicus*	Cichlidae	FW	H	0.206	22.50	65	3	10	Indiv
El-Sheekh (2014)	sp1	1.4373	4.90	0.14	4.59	0.27	30	30	*O. niloticus x O. mossambicus*	Cichlidae	FW	H	0.206	28.00	65	3	10	Indiv
Abdel-Latif (2014)	sp2	3.7009	0.16	0.04	0.04	0.00	20	20	*Oreochromis niloticus*	Cichlidae	FW	H	50.00	10.00	28	1	20	Indiv
Abdel-Latif (2014)	sp2	1.8504	0.10	0.04	0.04	0.00	20	20	*Oreochromis niloticus*	Cichlidae	FW	H	50.00	2.500	28	1	20	Indiv
Abdel-Latif (2014)	sp2	2.4673	0.12	0.04	0.04	0.00	20	20	*Oreochromis niloticus*	Cichlidae	FW	H	50.00	5.000	28	1	20	Indiv
Abdel-Tawwab (2009)	sp3	0.7812	2.62	0.10	2.41	0.36	60	60	*Oreochromis niloticus*	Cichlidae	FW	H	1.880	0.500	84	NA	20	Indiv
Abdel-Tawwab (2009)	sp3	0.1351	2.45	0.20	2.41	0.36	60	60	*Oreochromis niloticus*	Cichlidae	FW	H	1.880	0.125	84	NA	20	Indiv
Abdel-Tawwab (2009)	sp3	0.2653	2.49	0.22	2.41	0.36	60	60	*Oreochromis niloticus*	Cichlidae	FW	H	1.880	0.250	84	NA	20	Indiv
Abdel-Tawwab (2009)	sp3	0.1092	2.45	0.36	2.41	0.36	60	60	*Oreochromis niloticus*	Cichlidae	FW	H	1.880	0.750	84	NA	20	Indiv
Abdel-Tawwab (2009)	sp3	0.0793	2.44	0.39	2.41	0.36	60	60	*Oreochromis niloticus*	Cichlidae	FW	H	1.880	1.000	84	NA	20	Indiv
Belal (2012)	sp4	0.3523	2.05	0.51	1.87	0.51	40	40	*Oreochromis niloticus*	Cichlidae	FW	H	7.080	1.000	84	3	10	Indiv
Hussein (2013)	sp5	6.3402	5.85	0.08	5.04	0.16	39	39	*Oreochromis niloticus*	Cichlidae	FW	H	0.020	43.63	77	3	50	Batch
Mahmoud (2018)	sp6	0.2032	0.82	0.23	0.78	0.15	60	60	*Oreochromis niloticus*	Cichlidae	FW	H	9.300	1.000	83	3	20	Indiv
Mahmoud (2018)	sp6	− 0.4162	0.73	0.08	0.78	0.15	60	60	*Oreochromis niloticus*	Cichlidae	FW	H	9.300	2.000	83	3	20	Indiv
Khalila (2018)	sp7	0.2969	1.80	0.38	1.70	0.28	90	90	*Oreochromis niloticus*	Cichlidae	FW	H	3.780	0.500	84	3	10	Indiv
Hussein (2014)	sp8	4.4047	4.20	0.20	3.50	0.10	75	75	*Oreochromis niloticus*	Cichlidae	FW	H	30.00	21.80	63	3	50	Indiv
Leite (2019)	sp9	− 1.5181	4.57	0.32	5.12	0.31	4	4	*Oreochromis niloticus*	Cichlidae	SW	H	1.000	20.00	45	2	25	Batch
Teuling (2017)	sp10	0.4988	2.64	0.37	2.41	0.37	3	3	*Oreochromis niloticus*	Cichlidae	FW	H	37.40	30.00	33	3	35	Batch
Liu (2019)	sp11	0.1953	2.57	0.61	2.36	1.35	15	15	*Pelteobagrus fulvidraco*	Bagridae	FW	C	3.100	5.700	50	3	50	Batch
Liu (2019)	sp11	− 0.0359	2.32	0.73	2.36	1.35	15	15	*Pelteobagrus fulvidraco*	Bagridae	FW	C	3.100	11.50	50	3	50	Batch
Liu (2019)	sp11	− 0.0066	2.35	1.59	2.36	1.35	15	15	*Pelteobagrus fulvidraco*	Bagridae	FW	C	3.100	17.20	50	3	50	Batch
Liu (2019)	sp11	− 0.0388	2.29	2.08	2.36	1.35	15	15	*Pelteobagrus fulvidraco*	Bagridae	FW	C	3.100	23.00	50	3	50	Batch
Liu (2019)	sp11	− 0.4739	1.81	0.86	2.36	1.35	15	15	*Pelteobagrus fulvidraco*	Bagridae	FW	C	3.100	28.70	50	3	50	Batch
Yu (2018)	sp12	7.0039	1.69	0.05	1.40	0.03	90	90	*Plectropomus leopardus*	Serranidae	SW	C	18.00	10.00	56	3	30	Indiv
Yu (2018)	sp12	0.3906	1.41	0.02	1.40	0.03	90	90	*Plectropomus leopardus*	Serranidae	SW	C	18.00	2.000	56	3	30	Indiv
Yu (2018)	sp12	0.8241	1.45	0.08	1.40	0.03	90	90	*Plectropomus leopardus*	Serranidae	SW	C	18.00	4.000	56	3	30	Indiv
Yu (2018)	sp12	0.2415	1.41	0.05	1.40	0.03	90	90	*Plectropomus leopardus*	Serranidae	SW	C	18.00	6.000	56	3	30	Indiv
Yu (2018)	sp12	3.7787	1.58	0.06	1.40	0.03	90	90	*Plectropomus leopardus*	Serranidae	SW	C	18.00	8.000	56	3	30	Indiv
Rosas (2019)	sp13	12.3432	4.32	0.08	3.38	0.07	30	30	*Mugil liza*	Mugilidae	SW	O	0.470	22.50	90	4	25	Indiv
Rosas (2019)	sp13	15.9283	4.30	0.04	3.38	0.07	30	30	*Mugil liza*	Mugilidae	SW	O	0.470	15.00	90	4	25	Indiv
Rosas (2019)	sp13	8.1385	4.19	0.12	3.38	0.07	30	30	*Mugil liza*	Mugilidae	SW	O	0.470	30.00	90	4	25	Indiv
Rosas (2019)	sp13	3.3596	3.89	0.20	3.38	0.07	30	30	*Mugil liza*	Mugilidae	SW	O	0.470	45.00	90	4	25	Indiv
Adel (2016)	sp14	3.7430	2.78	0.19	2.22	0.09	60	60	*Huso huso*	Acipenseridae	FW	C	32.16	10.00	56	3	20	Indiv
Adel (2016)	sp14	1.1242	2.34	0.12	2.22	0.09	60	60	*Huso huso*	Acipenseridae	FW	C	32.16	2.500	56	3	20	Indiv
Adel (2016)	sp14	2.8706	2.56	0.14	2.22	0.09	60	60	*Huso huso*	Acipenseridae	FW	C	32.16	5.000	56	3	20	Indiv
Cao (2018)	sp15	0.7200	1.52	0.24	1.37	0.16	66	66	*Carassius auratus gibelio*	Cyprinidae	FW	O	15.37	3.380	46	3	22	Indiv
Cao (2018)	sp15	0.6240	1.50	0.24	1.37	0.16	66	66	*Carassius auratus gibelio*	Cyprinidae	FW	O	15.37	6.760	46	3	22	Indiv
Cao (2018)	sp15	− 0.1935	1.32	0.32	1.37	0.16	66	66	*Carassius auratus gibelio*	Cyprinidae	FW	O	15.37	13.52	46	3	22	Indiv
Rosas (2019)	sp16	0.4198	3.75	0.12	3.36	1.30	42	42	*Mugil liza*	Mugilidae	SW	O	0.260	1.950	80	3	14	Indiv
Rosas (2019)	sp16	0.1501	3.50	0.17	3.36	1.30	42	42	*Mugil liza*	Mugilidae	SW	O	0.260	1.200	80	3	14	Indiv
Rosas (2019)	sp16	0.0841	3.44	0.31	3.36	1.30	42	42	*Mugil liza*	Mugilidae	SW	O	0.260	2.700	80	3	14	Indiv
Rosas (2019)	sp16	− 0.7006	2.70	0.25	3.36	1.30	42	42	*Mugil liza*	Mugilidae	SW	O	0.260	3.900	80	3	14	Indiv
Ribeiro (2019)	sp17	1.5381	3.79	0.22	3.43	0.24	24	24	*C. macropomum x P. brachypomus*	Serrasalmidae	FW	H	3.560	40.00	64	3	8	Indiv
Ribeiro (2019)	sp17	0.9435	3.67	0.26	3.43	0.24	24	24	*C. macropomum x P.brachypomus*	Serrasalmidae	FW	H	3.560	20.00	64	3	8	Indiv
Nasir (2018)	sp18	0.4371	4.38	0.47	4.15	0.57	90	90	*Clarias gariepinus*	Clariidae	FW	O	2.620	3.000	90	3	30	Indiv
Nasir (2018)	sp18	0.0805	4.20	0.66	4.15	0.57	90	90	*Clarias gariepinus*	Clariidae	FW	O	2.620	1.000	90	3	30	Indiv
Nasir (2018)	sp18	0.1749	4.25	0.57	4.15	0.57	90	90	*Clarias gariepinus*	Clariidae	FW	O	2.620	5.000	90	3	30	Indiv
Nasir (2018)	sp18	0.3381	4.36	0.66	4.15	0.57	90	90	*Clarias gariepinus*	Clariidae	FW	O	2.620	7.000	90	3	30	Indiv
Chainapong (2018)	sp19	0.0084	1.82	1.59	1.80	2.94	30	30	*Clarias macrocephalus*	Clariidae	FW	O	19.00	10.00	120	3	50	Batch
Chainapong (2018)	sp19	− 0.0254	1.70	4.65	1.80	2.94	30	30	*Clarias macrocephalus*	Clariidae	FW	O	19.00	5.000	120	3	50	Batch
El-Ward (2016)	sp20	5.6908	1.87	0.11	1.24	0.11	60	60	*Oreochromis niloticus*	Cichlidae	FW	H	9.900	10.89	56	3	20	Indiv
El-Ward (2016)	sp20	− 0.7769	1.15	0.12	1.24	0.11	60	60	*Oreochromis niloticus*	Cichlidae	FW	H	9.900	2.730	56	3	20	Indiv
El-Ward (2016)	sp20	1.2087	1.40	0.15	1.24	0.11	60	60	*Oreochromis niloticus*	Cichlidae	FW	H	9.900	5.450	56	3	20	Indiv
El-Ward (2016)	sp20	4.1041	1.76	0.14	1.24	0.11	60	60	*Oreochromis niloticus*	Cichlidae	FW	H	9.900	8.170	56	3	20	Indiv
Zeinab (2019)	sp21	0.2463	2.32	0.40	2.22	0.40	45	45	*Oreochromis niloticus*	Cichlidae	FW	H	2.700	3.000	95	3	15	Indiv
Zeinab (2019)	sp21	0.0000	2.22	0.40	2.22	0.40	45	45	*Oreochromis niloticus*	Cichlidae	FW	H	2.700	5.000	95	3	15	Indiv
Zeinab (2019)	sp21	− 0.2463	2.12	0.40	2.22	0.40	45	45	*Oreochromis niloticus*	Cichlidae	FW	H	2.700	7.000	95	3	15	Indiv
Teimouri (2016)	sp22	0.2638	1.39	0.30	1.31	0.30	36	36	*Oncorhynchus mykiss*	Salmonidae	FW	C	101.0	7.500	70	3	12	Indiv
Teimouri (2016)	sp22	0.0800	1.33	0.18	1.31	0.30	36	36	*Oncorhynchus mykiss*	Salmonidae	FW	C	101.0	2.500	70	3	12	Indiv
Teimouri (2016)	sp22	− 0.1999	1.26	0.18	1.31	0.30	36	36	*Oncorhynchus mykiss*	Salmonidae	FW	C	101.0	5.000	70	3	12	Indiv
Teimouri (2016)	sp22	0.1977	1.39	0.48	1.31	0.30	36	36	*Oncorhynchus mykiss*	Salmonidae	FW	C	101.0	10.00	70	3	12	Indiv
Güroy (2019)	sp23	− 2.7770	0.81	0.05	0.98	0.07	60	60	*Oncorhynchus mykiss*	Salmonidae	FW	C	135.0	4.000	84	3	20	Indiv
El-Murr (2014)	sp24	0.5289	0.57	0.39	0.40	0.23	60	60	*Oreochromis niloticus*	Cichlidae	FW	H	33.00	1.500	60	3	50	Indiv
El-Murr (2014)	sp24	0.2177	0.46	0.31	0.40	0.23	60	60	*Oreochromis niloticus*	Cichlidae	FW	H	33.00	0.500	60	3	50	Indiv
El-Murr (2014)	sp24	0.4978	0.56	0.39	0.40	0.23	60	60	*Oreochromis niloticus*	Cichlidae	FW	H	33.00	1.000	60	3	50	Indiv
Al-Zayat (2019)	sp25	4.3783	1.26	0.05	1.05	0.04	20	20	*Oreochromis niloticus*	Cichlidae	FW	H	6.000	0.750	60	2	10	Indiv
Al-Zayat (2019)	sp25	0.2255	1.08	0.18	1.05	0.04	20	20	*Oreochromis niloticus*	Cichlidae	FW	H	6.000	0.250	60	2	10	Indiv
Al-Zayat (2019)	sp25	1.5341	1.12	0.04	1.05	0.04	20	20	*Oreochromis niloticus*	Cichlidae	FW	H	6.000	0.500	60	2	10	Indiv
Roohani (2019)	sp26	0.4656	1.58	1.75	0.81	1.42	12	12	*Salmo trutta caspius*	Salmonidae	FW	C	11.00	3.960	70	3	40	Batch
Roohani (2019)	sp26	0.2204	1.10	1.10	0.81	1.42	12	12	*Salmo trutta caspius*	Salmonidae	FW	C	11.00	1.320	70	3	40	Batch
Roohani (2019)	sp26	0.2466	1.16	1.31	0.81	1.42	12	12	*Salmo trutta caspius*	Salmonidae	FW	C	11.00	2.640	70	3	40	Batch
Roohani (2019)	sp26	0.2487	1.39	2.85	0.81	1.42	12	12	*Salmo trutta caspius*	Salmonidae	FW	C	11.00	5.280	70	3	40	Batch
Kermani (2020)	sp27	1.9740	3.10	0.10	2.90	0.10	30	30	*Oncorhynchus mykiss*	Salmonidae	FW	C	12.60	0.025	56	3	10	Indiv
Kermani (2020)	sp27	0.0000	2.90	0.50	2.90	0.10	30	30	*Oncorhynchus mykiss*	Salmonidae	FW	C	12.60	0.050	56	3	10	Indiv
Kermani (2020)	sp27	− 0.6242	2.80	0.20	2.90	0.10	30	30	*Oncorhynchus mykiss*	Salmonidae	FW	C	12.60	0.100	56	3	10	Indiv
Kermani (2020)	sp27	− 1.9740	2.70	0.10	2.90	0.10	30	30	*Oncorhynchus mykiss*	Salmonidae	FW	C	12.60	0.250	56	3	10	Indiv
Gouveia (2003) (koicarp)	sp28	0.0000	0.20	0.10	0.20	0.10	25	25	*Cyprinus carpio*	Cyprinidae	FW	O	24.60	4.000	70	2	25	Indiv
Gouveia (2003) (goldfish)	sp29	0.0000	1.40	0.04	1.40	0.09	25	25	*Carassius auratus*	Cyprinidae	FW	O	0.900	4.000	70	2	25	Indiv
Abdel-Warith (2019)	sp30	− 0.0322	1.89	0.73	1.91	0.47	30	30	*Oreochromis niloticus*	Cichlidae	FW	H	15.98	4.000	84	2	15	Indiv
Abdel-Warith (2019)	sp30	− 0.2929	1.80	0.23	1.91	0.47	30	30	*Oreochromis niloticus*	Cichlidae	FW	H	15.98	8.000	84	2	15	Indiv
Abdel-Warith (2019)	sp30	− 0.1902	1.83	0.35	1.91	0.47	30	30	*Oreochromis niloticus*	Cichlidae	FW	H	15.98	12.00	84	2	15	Indiv
Khanzadeh (2016)	sp31	0.6901	2.31	0.10	2.20	0.20	48	48	*Trichopodus trichopterus*	Osphronemidae	FW	H	1.290	10.00	112	3	48	Indiv
Khanzadeh (2016)	sp31	− 0.2480	2.15	0.20	2.20	0.20	48	48	*Trichopodus trichopterus*	Osphronemidae	FW	H	1.290	2.500	112	3	48	Indiv
Khanzadeh (2016)	sp31	0.0778	2.22	0.30	2.20	0.20	48	48	*Trichopodus trichopterus*	Osphronemidae	FW	H	1.290	5.000	112	3	48	Indiv
Khanzadeh (2016)	sp31	0.2480	2.25	0.20	2.20	0.20	48	48	*Trichopodus trichopterus*	Osphronemidae	FW	H	1.290	20.00	112	3	48	Indiv
Raji (2019)	sp32	0.3338	2.29	0.09	2.26	0.09	80	80	*Clarias gariepinus*	Clariidae	FW	O	41.86	18.70	56	3	10	Indiv
Raji (2019)	sp32	0.2225	2.28	0.09	2.26	0.09	80	80	*Clarias gariepinus*	Clariidae	FW	O	41.86	12.50	56	3	10	Indiv
Viswanathan (2019)	sp33	0.1114	1.90	2.00	1.70	1.50	25	25	*Cyprinus carpio*	Cyprinidae	FW	O	21.50	15.00	28	3	25	Indiv
Viswanathan (2019)	sp33	0.0772	1.80	1.00	1.70	1.50	25	25	*Cyprinus carpio*	Cyprinidae	FW	O	21.50	5.000	28	3	25	Indiv
Viswanathan (2019)	sp33	0.0656	1.80	1.50	1.70	1.50	25	25	*Cyprinus carpio*	Cyprinidae	FW	O	21.50	10.00	28	3	25	Indiv
Viswanathan (2019)	sp33	0.0656	1.80	1.50	1.70	1.50	25	25	*Cyprinus carpio*	Cyprinidae	FW	O	21.50	20.00	28	3	25	Indiv
Viswanathan (2019)	sp33	0.0000	1.70	1.00	1.70	1.50	25	25	*Cyprinus carpio*	Cyprinidae	FW	O	21.50	25.00	28	3	25	Indiv
Kim (2013)	sp34	4.4363	0.81	0.01	0.68	0.04	75	75	*Oplegnathus fasciatus*	Oplegnathidae	SW	H	57.00	9.000	56	3	25	Indiv
Kim (2013)	sp34	1.8877	0.74	0.02	0.68	0.04	75	75	*Oplegnathus fasciatus*	Oplegnathidae	SW	H	57.00	18.00	56	3	25	Indiv
Kim (2013)	sp34	0.1951	0.69	0.06	0.68	0.04	75	75	*Oplegnathus fasciatus*	Oplegnathidae	SW	H	57.00	26.00	56	3	25	Indiv
Rosas (2019)	sp35	6.3017	4.21	0.17	3.38	0.07	30	30	*Mugil liza*	Mugilidae	SW	O	0.470	4.000	75	3	10	Indiv
Rosas (2019)	sp35	6.1543	4.15	0.16	3.38	0.07	30	30	*Mugil liza*	Mugilidae	SW	O	0.470	2.000	75	3	10	Indiv
Siringi (2007)	sp36	2.1807	0.52	0.08	0.35	0.08	60	60	*Oreochromis shiranus*	Cichlidae	FW	H	5.860	0.700	70	3	20	Indiv
Siringi (2007)	sp36	1.0262	0.43	0.08	0.35	0.08	60	60	*Oreochromis shiranus*	Cichlidae	FW	H	5.860	0.350	70	3	20	Indiv
Siringi (2007)	sp36	1.0547	0.48	0.15	0.35	0.08	60	60	*Oreochromis shiranus*	Cichlidae	FW	H	5.860	1.050	70	3	20	Indiv

*SMD* standardized mean difference, *Me* mean SGR of experimental group, *Se* standard deviation of SGR of experimental group, *Mc* mean SGR of control group, *Sc* standard deviation of SGR of control group, *Ne* sample size of experimental group, *Nc* sample size of control group, Hab. *FW* freshwater, *SW* sea water, *Diet* Carnivore (C), Herbivore (H), Omnivore (O), *Size* initial mass (g), *Replac. %* Spirulina replacement level (%), *Days* duration of trial (days), *Tanks* number of replicate tanks, *Dens.* tank density (No. fish/tank), *Data* type of measurement (individual weights or batch weighing).

#### Effect sizes

**Figure 2:**
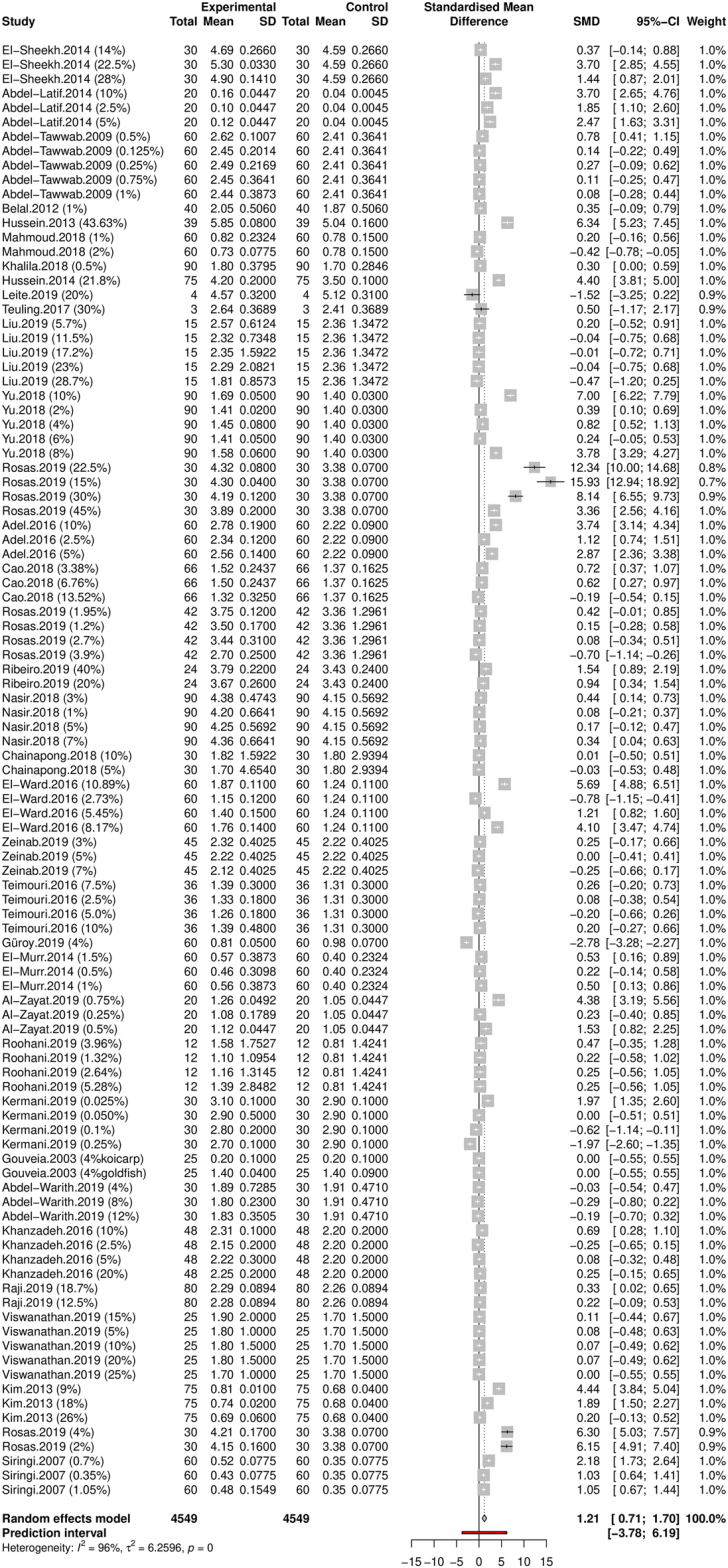
Forest plot summarizing the effect of *Spirulina* replacement on the Specific Growth Rate (SGR) of farmed finfish. Each trial (n = 101) is represented by a square whose size is proportional to its relative weight, its width represents the 95% CI, the horizontal line the 99% CI, and the center the Standardized Mean Difference (SMD) corrected for small sample size (Hedge’s *g*). The grey diamond at the bottom represents the overall effect extending over the 95% CI. The solid vertical line denotes the zero effect, and the dotted vertical line the SMD under a random effects model.

Standardized mean differences (SMD), corrected for small sample sizes, varied between − 2.78 and 15.93. The pooled SMD of the random effects model was 1.21 (95% CI 0.71–1.70), which was significantly different from zero (*t* = 4.83, *P* < 0.001), and indicated that *Spirulina* inclusion in the diet had a positive effect on fish growth (Fig. 2). However, heterogeneity between studies was very high (*Q* = 2732, *df* = 100, *P* < 0.001; *I*^2^ = 96.3% *τ*^2^ = 6.26) and the prediction interval was wide (95% CI 3.78–6.16), indicating that negative effects on growth cannot be ruled out in future studies. Just over 48% of control-treatment comparisons (49/101) were statistically significant, involving 19 of the 36 independent studies (53%). The average replacement of fish meal with *Spirulina* that yielded an improvement in SGR was 8.42% (SD = 10.26), but enhanced growth was detected with *Spirulina* replacement as low as 0.025% in rainbow trout (*Oncorhynchus mykiss*)^45^.

#### Dose-dependent effects

**Figure 3:**
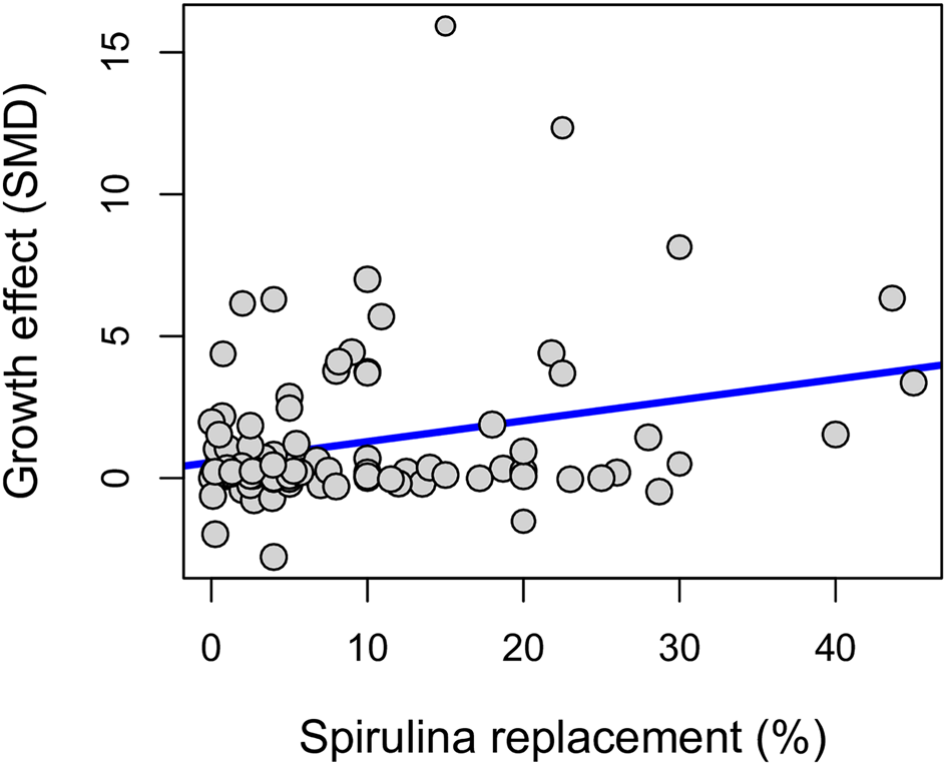
Bubble plot showing the estimated regression slope of the meta-regression on the effect of *Spirulina* replacement (%) on the Standardized Mean Difference in Specific Growth Rate (%). The size of the points is proportional to the weight of each study.

Results of meta-regression by mixed-effects modelling indicates that there is a significant positive relationship between *Spirulina* replacement level and specific growth rate while statistically controlling for variation among fish families (*F*_11,89_ = 4.629, *P* < 0.001; Fig. 3). The minimal adequate model included *Spirulina* replacement and family as the only significant predictors of changes in specific growth rate. Initial size (*t* = − 0.685, *P* = 0.495) and habitat (*t* = − 1.754, *P* = 0.083) were not significant, while feeding guild was redundant, and were dropped from the full main effects model.

A 1% increase in *Spirulina* inclusion is expected to result in a 0.07% mean increase in SGR (95% CI 0.03–0.12%; Fig. 3), although the model only accounted for 29.4% of the observed heterogeneity and the amount of residual heterogeneity was high (*Q*_E_ = 2143.6, *df* = 89, *P* < 0.001). Inspection of estimates indicated that negative impacts were also possible. Two families, Bagridae (*t* = − 2.277, *P* = 0.025) and Cyprinidae (*t* = − 2.043, *P* = 0.044) deviated significantly from the general trend and showed a reduction in growth with increasing *Spirulina* replacement levels, while one family, Salmonidae, showed a near significant negative effect (*t* = − 1.909, *P* = 0.059).

#### Validity of results

**Figure 4:**
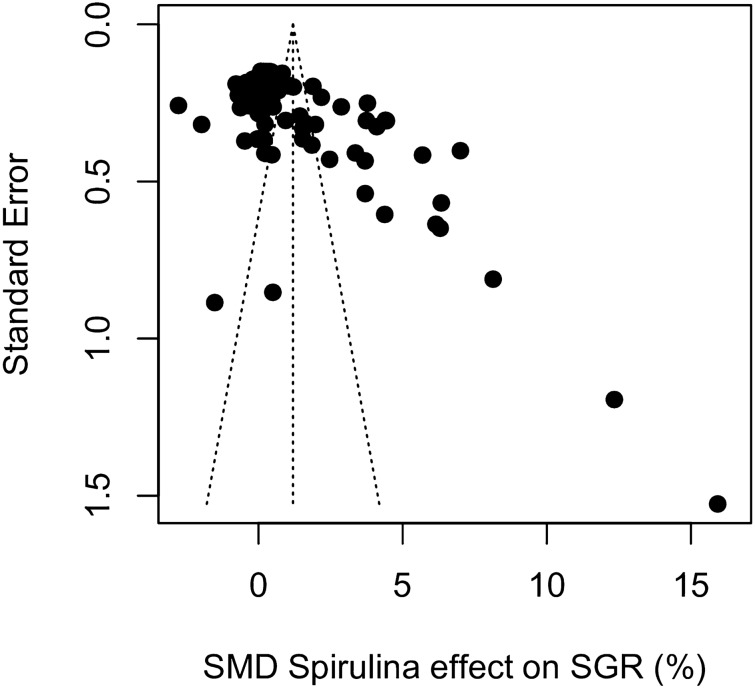
Funnel plot showing the relationship between the SMD and the standard error (inverted scale) for the effects of *Spirulina* replacement on specific growth rate (SGR). Each point represents a treatment–control comparison and the dotted vertical line denotes the global SMD under a random effects model. An asymmetric distribution of points outside the funnel might be indicative of publication bias.

A strong asymmetry was observed in the funnel plot (Fig. 4), which might be indicative of publication bias. Several studies reporting large effects were more precise than one might expect and clustered at the bottom right corner, far outside the boundaries of the funnel. A linear regression test of funnel plot asymmetry (Egger’s test) confirmed the observed asymmetry (*t*_99_ = 5.37, *P* < 0.001; bias coefficient = 7.04, SE = 1.31). However, caution must be exercised as the high level of heterogeneity in the data set likely also contributed to the asymmetry observed in the funnel plot.

Results from the *P*-curve analysis indicated that the distribution of significant results was significantly right skewed according to all three tests (*P* binomial < 0.001, full curve *P* < 0.001; half curve *P* < 0.001), while results from the flatness test could not reject the hypothesis that the distribution of significant results was dependent on the significance level (*P* binomial > 0.999, full curve *P* > 0.999; half curve *P* > 0.999). Overall, the evidential value suggests that the observed results are driven by a true underlying effect and do not appear to have been affected by publication bias in the form of *P*-hacking.

**Figure 5:**
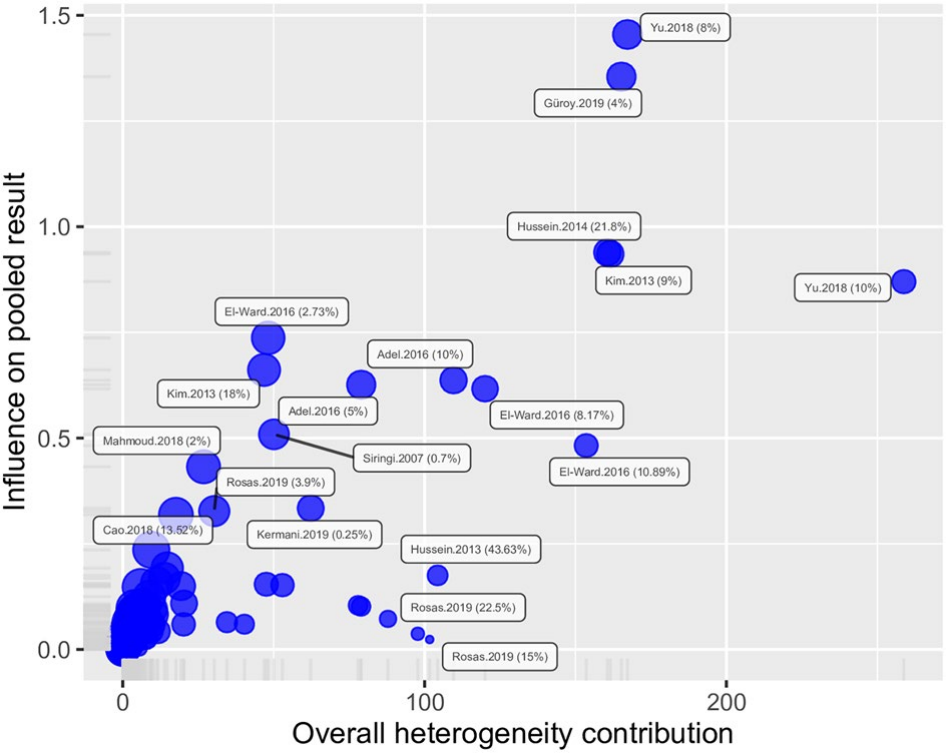
Baujat bubble plot used to identify potential outliers in the *Spirulina* data set, showing the contribution of each study to the overall heterogeneity and its influence under a random effects model. The size of each point is proportional to its relative weight in the meta-analysis. Five trials in the upper right corner accounted for a large share of the observed heterogeneity and were also overly influential which merited further scrutiny.

Inspection of Baujat diagnostic plots detected five results which were overly influential (two from the same study) and which also contributed greatly to the overall heterogeneity (Fig. 5), while formal outlier analysis detected 70 extreme results. Reanalysis of the data without the overly influential points resulted in a pooled SMD of 1.09 (95% CI 0.60–1.57) which is still significantly different from zero (*t* = 4.44, *P* < 0.001). Similarly, removal of outliers resulted in a statistically significant SMD of 0.86 (95% CI 0.64–1.07; *t* = 8.14, *P* < 0.001). These results indicated a significant positive effect of *Spirulina* on fish growth which was robust to the presence of extreme values, although heterogeneity even without outliers continued to be high (*I*^2^ = 76.8%, *Q* = 129.6, *df* = 30, *P* < 0.001) suggesting that there were underlying structural differences between studies beyond sampling error.

#### Subgroup analysis

To gain insights into the sources of heterogeneity, we conducted a subgroup analysis. Significant differences in *Spirulina* effects were found with respect to fish family (*Q* = 53.42, *df* = 10, *P* < 0.001) and habitat (*Q* = 7.11, *df* = 1, *P* = 0.008), but not with respect to feeding guild (*P* = 0.305) or type of measurements (*P* = 0.098; Table 2). Of the 11 fish families examined, three families (Cichlidae, Clariidae and Mugilidae) showed a statistically significant increase on growth, this effect being strongest for Mugilidae (SMD = 5.11; 95% CI 1.09–9.12), followed by Cichlidae (SMD = 1.20; 95% CI 0.60–1.80) and Clariidae (SMD = 0.23; 95% CI 0.10–0.36).

**Table 2:** Sources of heterogeneity and subgroup analysis in the *Spirulina* dataset according to a random effects model.

Grouping	*k*	SMD	95% CI	*Q*	*I*^*2*^
Family					
Acipenseridae	3	2.56	[− 0.753; 5.884]	61.7	96.8%
Bagridae	5	-0.07	[− 0.372; 0.232]	1.8	0.0%
Cichlidae*	38	1.20	[0.605; 1.802]	942.5	96.1%
Clariidae*	8	0.23	[0.100; 0.357]	5.7	0.0%
Cyprinidae	10	0.18	[− 0.054; 0.412]	21.3	57.7%
Mugilidae*	10	5.11	[1.089; 9.121]	522.6	98.3%
Oplegnathidae	3	2.16	[− 3.135; 7.459]	158.3	98.7%
Osphronemidae	4	0.19	[− 0.430; 0.810]	10.6	71.7%
Salmonidae	13	0.15	[− 0.859; 0.550]	194.5	93.8%
Serranidae	5	2.43	[− 1.192; 6.055]	388.3	99.0%
Serrasalmidae	2	1.23	[− 2.546; 5.001]	1.7	42.4%
Test for subgroup differences			*Q* = 53.42, *df* = 10, *P* < 0.001		
Habitat					
Freshwater*	82	0.705	[0.381; 1.022]	1531.2	94.7%
Marine*	19	3.557	[1.336; 5.777]	1092.0	94.8%
Test for subgroup differences			*Q* = 7.11, *df* 1, *P* = 0.008		
Feeding					
Carnivores	26	0.680	[− 0.094; 1.454]	936.2	97.3%
Omnivores*	28	1.862	[0.327; 3.396]	576.9	95.3%
Herbivores*	47	1.177	[0.667; 1.687]	1181.7	96.1%
Test for subgroup differences			*Q* = 2.37, *df* 2, *P* = 0.305		
Measurement					
Individual data*	87	1.322	[0.776; 1.888]	2596.1	96.7%
Batch data	14	0.436	[− 0.566; 1.437]	126.8	89.8%
Test for subgroup differences			*Q* = 2.74, *df* = 1, *P* = 0.098		

Groups that display a positive effect of *Spirulina* on specificgrowth rate are denoted by an asterisk.

*k* number of studies, *SMD* standardized mean difference compared to controls, *95% CI* 95% confidence interval around SMD, *Q* Cochran’s measure of heterogeneity, *I*^*2*^ percentage of variability unaccounted by sampling error.

Differences were also found between freshwater and marine species, both displaying a significant increase in growth following *Spirulina* inclusion, the positive effect on growth being ~ 5 times greater in marine fish (SMD = 3.56; 95% CI 1.34–5.78) than in freshwater fish (SMD = 0.70; 95% CI 0.38–1.02). Significant *Spirulina* benefits on growth were found for omnivores and herbivores, but not for carnivores. Studies that weighed fish individually were also more likely to reveal a positive effect of *Spirulina* on growth than those which used batch weighing (Table 2).

Although the subgroup analysis uncovered some of the sources of variation, substantial heterogeneity persisted both between and within groups. Six families (Acipenseridae, Cichlidae, Mugilidae, Oplegnathidae, Salmonidae, Serranidae).

showed substantial heterogeneity (*I*^2^ > 75%), three families showed moderate heterogeneity (*I*^2^ = 25–75%; Cyprinidae, Osphronemidae, Serrasalmidae) and only two families displayed modest heterogeneity (*I*^2^ < 25%; Bagridae, Clariidae). Variation among habitats, feeding guilds, and types of measurement were all substantial and not markedly different from the overall level of heterogeneity observed in the entire data set (*I*^2^ = 96%). This suggests that other sources of variation were at play beyond those that could be accounted for in the analysis.

### Effects of *Schizochytrium* replacement on fillet omega-3 content

We found 14 quantitative studies on the effects of *Schizochytrium* replacement on omega-3 fillet content, representing *k* = 43 control-treatment comparisons, that met the selection criteria. *Schizochytrium* studies were carried out in 10 species belonging to 9 different fish families. Study subjects ranged in size between 0.02 g and 850 g (mean = 65.7 g, SD = 183.3) and consisted of both juveniles and adults of marine and freshwater species in equal measure, although most results referred to carnivorous species (65%), such as Atlantic salmon (*Salmo salar*—21% of studies) and red drum (*Sciaenops ocellatus*—14% of studies). In most cases (79%), studies were carried out in triplicate tanks and involved an average of 132 individuals per tank (SD = 371), with feeding trials lasting between 21 and 133 days (mean = 64 days, SD = 25.8; Table 3). Replacement levels of *Schizochytrium* varied from 2 to 100% (mean = 42.6%, SD = 30.8).

**Table 3:** Results of feeding studies assessing the effects of *Schizochytrium* replacement on the omega-3 content of the fish fillet.

Author	Study ID	SMD	Me	Se	Mc	Sc	Ne	Nc	Species	Family	Hab	Diet	Size (g)	Replac. %	Days	Tanks	Dens	Data
Ortega (2016)	sc1	1.3714	33.30	4.67	25.30	4.67	3	3	*Epinephelus lanceolatus*	Serranidae	SW	C	45.9	100	84	3	6	Batch
Ortega (2016)	sc1	1.4228	33.60	4.67	25.30	4.67	3	3	*Epinephelus lanceolatus*	Serranidae	SW	C	45.9	100	84	3	6	Batch
Ortega (2016)	sc1	0.7371	29.60	4.67	25.30	4.67	3	3	*Epinephelus lanceolatus*	Serranidae	SW	C	45.9	68	84	3	6	Batch
Ganuza (2008)	sc2	− 0.3179	30.90	21.48	38.60	26.33	48	48	*Sparus aurata*	Sparidae	SW	C	0.15	100	21	3	NA	Indiv
Li (2009)	sc3	0.2896	4.12	4.76	2.73	4.76	50	50	*Ictalurus punctatus*	Ictaluridae	FW	O	20.4	10	63	5	15	Indiv
Li (2009)	sc3	0.5563	5.40	4.76	2.73	4.76	50	50	*Ictalurus punctatus*	Ictaluridae	FW	O	20.4	25	63	5	15	Indiv
Li (2009)	sc3	0.5229	5.24	4.76	2.73	4.76	50	50	*Ictalurus punctatus*	Ictaluridae	FW	O	20.4	40	63	5	15	Indiv
Li (2009)	sc3	0.7625	6.39	4.76	2.73	4.76	50	50	*Ictalurus punctatus*	Ictaluridae	FW	O	20.4	50	63	5	15	Indiv
Velazquez (2018)	sc4	− 1.9846	15.47	0.05	16.46	0.67	9	9	*Sciaenops ocellatus*	Sciaenidae	SW	C	2.3	10	42	3	20	Indiv
Velazquez (2018)	sc4	− 3.8690	14.53	0.05	16.46	0.67	9	9	*Sciaenops ocellatus*	Sciaenidae	SW	C	2.3	20	42	3	20	Indiv
Velazquez (2018)	sc4	− 3.9550	14.09	0.45	16.46	0.67	9	9	*Sciaenops ocellatus*	Sciaenidae	SW	C	2.3	30	42	3	20	Indiv
Velazquez (2018)	sc4	3.1414	18.72	0.70	16.46	0.67	9	9	*Sciaenops ocellatus*	Sciaenidae	SW	C	2.3	40	42	3	20	Indiv
Velazquez (2018)	sc4	− 1.1499	14.74	1.90	16.46	0.67	9	9	*Sciaenops ocellatus*	Sciaenidae	SW	C	2.3	50	42	3	20	Indiv
Velazquez (2018)	sc4	11.2993	22.22	0.15	16.46	0.67	9	9	*Sciaenops ocellatus*	Sciaenidae	SW	C	2.3	100	42	3	20	Indiv
Velazquez (2019)	sc5	− 1.9048	15.50	0.10	16.50	0.70	9	9	*M. crhysops* × *M. saxatilis*	Moronidae	FW	C	10.6	10	42	3	12	Indiv
Velazquez (2019)	sc5	− 3.8095	14.50	0.10	16.50	0.70	9	9	*M. crhysops* × *M. saxatilis*	Moronidae	FW	C	10.6	20	42	3	12	Indiv
Velazquez (2019)	sc5	− 3.7577	14.10	0.50	16.50	0.70	9	9	*M. crhysops* × *M. saxatilis*	Moronidae	FW	C	10.6	30	42	3	12	Indiv
Velazquez (2019)	sc5	2.9932	18.70	0.70	16.50	0.70	9	9	*M. crhysops* × *M. saxatilis*	Moronidae	FW	C	10.6	40	42	3	12	Indiv
Velazquez (2019)	sc5	− 1.1973	14.70	1.90	16.50	0.70	9	9	*M. crhysops* × *M. saxatilis*	Moronidae	FW	C	10.6	50	42	3	12	Indiv
Sprague (2015)	sc6	− 0.5111	17.70	3.40	20.40	6.00	6	6	*Salmo salar*	Salmonidae	SW	C	850	7.7	133	3	130	Indiv
Sprague (2015)	sc6	− 0.2632	18.90	4.40	20.40	6.00	6	6	*Salmo salar*	Salmonidae	SW	C	850	21.1	133	3	130	Indiv
Miller.2007 FOc	sc7	− 2.3290	19.90	3.39	28.20	3.39	9	9	*Salmo salar*	Salmonidae	SW	C	40.0	20	63	3	24	Indiv
Miller (2007) FOc	sc7	1.7392	33.10	1.70	28.20	3.39	9	9	*Salmo salar*	Salmonidae	SW	C	40.0	100	63	3	24	Indiv
Miller (2007) POc	sc7	0.1969	19.90	3.39	18.90	5.94	9	9	*Salmo salar*	Salmonidae	SW	C	40.0	20	63	3	24	Indiv
Miller (2007) POc	sc7	3.0961	33.10	1.70	18.90	5.94	9	9	*Salmo salar*	Salmonidae	SW	C	40.0	100	63	3	24	Indiv
Luo (2018)	sc8	0.0038	14.50	73.97	14.10	126.39	60	60	*Acipenser baerii*	Acipenseridae	FW	C	0.02	20	30	3	800	Batch
Luo (2018)	sc8	0.0584	22.80	167.06	14.10	126.39	60	60	*Acipenser baerii*	Acipenseridae	FW	C	0.02	26.6	30	3	800	Batch
Luo (2018)	sc8	0.0544	24.00	222.41	14.10	126.39	60	60	*Acipenser baerii*	Acipenseridae	FW	C	0.02	33.3	30	3	800	Batch
Hoestenberghe (2016) FOc	sc9	5.4713	5.70	0.30	4.00	0.30	12	12	*Scortum barcoo*	Terapontidae	FW	O	9.98	27.3	70	3	50	Indiv
Hoestenberghe (2016) POc	sc9	10.6038	5.70	0.30	2.90	0.20	12	12	*Scortum barcoo*	Terapontidae	FW	O	9.98	27.3	70	3	50	Indiv
dos Santos (2019)	sc10	1.9676	2.20	0.07	1.80	0.24	4	4	*Oreochromis niloticus*	Cichlidae	FW	H	1.33	10	NA	4	12	Indiv
dos Santos (2019)	sc10	8.0407	3.50	0.10	1.80	0.24	4	4	*Oreochromis niloticus*	Cichlidae	FW	H	1.33	20	NA	4	12	Indiv
dos Santos (2019)	sc10	10.6223	4.20	0.14	1.80	0.24	4	4	*Oreochromis niloticus*	Cichlidae	FW	H	1.33	30	NA	4	12	Indiv
dos Santos (2019)	sc10	15.5822	5.20	0.12	1.80	0.24	4	4	*Oreochromis niloticus*	Cichlidae	FW	H	1.33	40	NA	4	12	Indiv
Seong (2019)	sc11	− 0.7671	12.00	0.80	13.40	2.40	20	20	*Pagrus major*	Sparidae	SW	C	8.80	63.6	84	2	20	Batch
Kousoulaki (2015)	sc12	0.3614	20.87	4.09	19.35	4.09	15	15	*Salmo salar*	Salmonidae	SW	C	213	2	84	3	40	Batch
Kousoulaki (2015)	sc12	0.7490	22.50	4.09	19.35	4.09	15	15	*Salmo salar*	Salmonidae	SW	C	213	13.4	84	3	40	Batch
Kousoulaki (2015)	sc12	− 0.2449	18.32	4.09	19.35	4.09	15	15	*Salmo salar*	Salmonidae	SW	C	213	33.3	84	3	40	Batch
Eryalçn (2015)	sc13	− 7.5500	26.75	0.04	29.45	0.48	9	9	*Sparus aurata*	Sparidae	SW	C	0.02	75	90	3	2100	Indiv
Sarker (2016)	sc14	0.0139	26.90	39.44	26.50	3.29	15	15	*Oreochromis niloticus*	Cichlidae	FW	H	1.52	25	84	3	40	Indiv
Sarker (2016)	sc14	0.0825	27.00	7.67	26.50	3.29	15	15	*Oreochromis niloticus*	Cichlidae	FW	H	1.52	50	84	3	40	Indiv
Sarker (2016)	sc14	− 0.0609	24.70	40.53	26.50	3.29	15	15	*Oreochromis niloticus*	Cichlidae	FW	H	1.52	75	84	3	40	Indiv
Sarker (2016)	sc14	− 0.0291	26.10	18.62	26.50	3.29	15	15	*Oreochromis niloticus*	Cichlidae	FW	H	1.52	100	84	3	40	Indiv

*SMD* standardized mean difference, *Me* mean omega-3 content of experimental group, *Se* standard deviation of omega-3 content of experimental group, *Mc* mean omega-3 content of control group, *Sc* standard deviation of omega-3 content of control group, *Ne* sample size of experimental group, *Nc* sample size of control group, Hab., *FW* freshwater, *SW* sea water, *Diet* Carnivore (C), Herbivore (H), Omnivore (O), *Size* Initial mass (g), *Replac. %* % *Schizochytrium* replacement level of fish oil (FO) or plan oil (PO), *Days* duration of trial (days), *Tanks* number of replicate tanks, *Dens.* tank density (No. fish/tank), *Data* type of measurement (individual measurements or batch measurement).

#### Effect sizes

**Figure 6:**
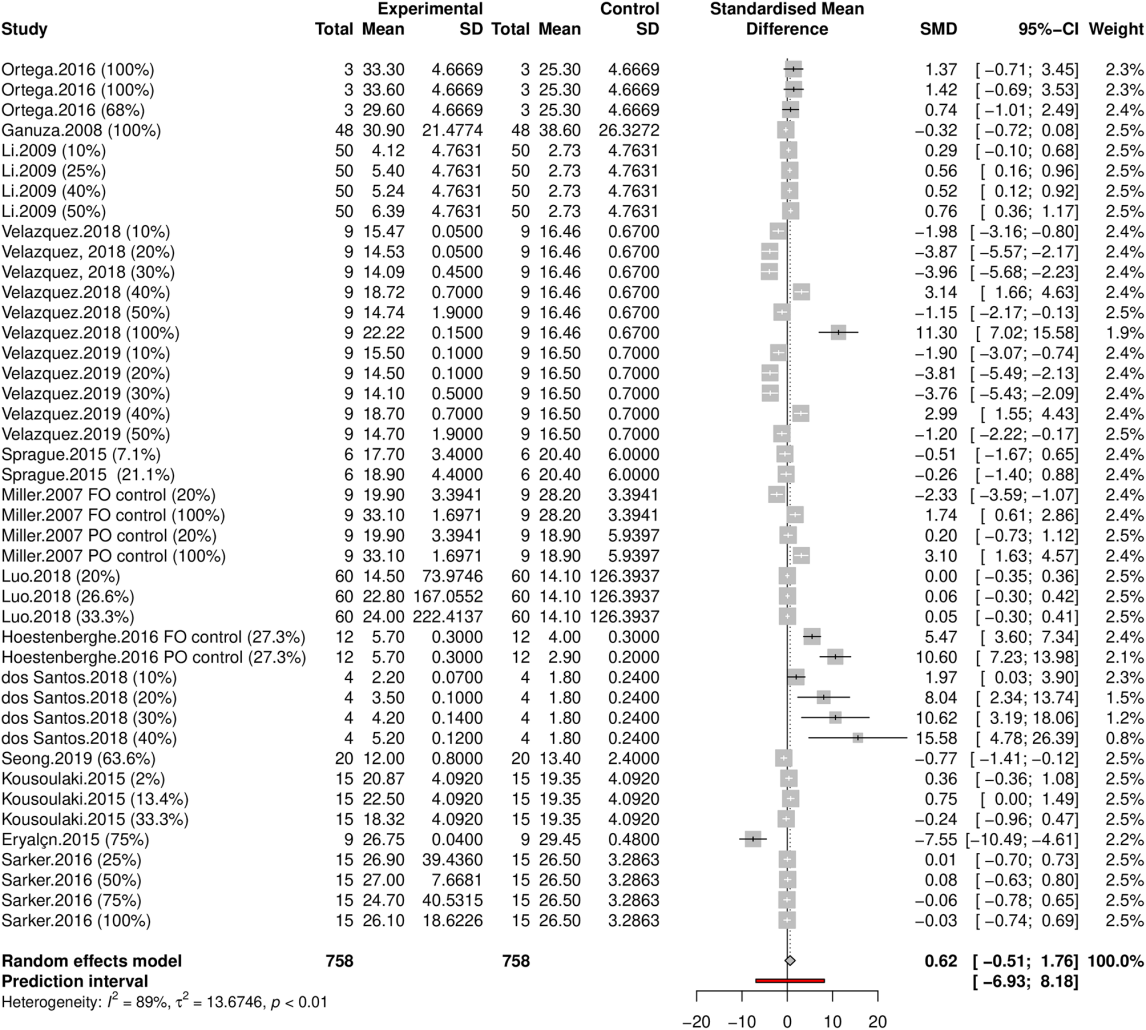
Forest plot summarizing the effect of *Schizochytrium* replacement on the omega-3 fillet content of farmed finfish. Each trial (n = 43) is represented by a square whose size is proportional to its relative weight, its width represents the 95% CI, the horizontal line the 99% CI, and the center the Standardized Mean Difference (SMD) corrected for small sample size (Hedge’s *g*). The grey diamond at the bottom represents the overall effect extending over the 95% CI. The solid vertical line denotes the zero effect, and the dotted vertical line the SMD under a random effects model.

Standardized mean differences (SMD), corrected for small sample sizes, varied between − 7.6 and 15.6 and resulted in a pooled SMD of 0.621 (95% CI − 0.51–1.76) which is not significantly different from zero (*t* = 1.11, *P* = 0.274), and indicated that *Schizochytrium* inclusion in the diet maintained the omega-3 fillet content (Fig. 6). However, as with results for *Spirulina,* heterogeneity between studies was very high (*Q* = 37.7, *df* = 42, *P* < 0.001; *I*^2^ = 88.9 *τ*^2^ = 13.67) and the prediction interval was very wide (95% CI 6.93–8.18), indicating that both negative and positive effects on omega-3 fillet content are possible. Over 60% of control-treatment comparisons (23/43) were statistically different from zero, involving 6 of the 14 independent studies (43%). The average replacement of fish and plant oil with *Schizochytrium* oil that yielded an improvement in omega-3 fillet content was 16.2% (SD = 21.1), but positive effects were reported with *Schizochytrium* replacement as low as 10% in Nile tilapia^46^.

#### Dose-dependent effects

**Figure 7:**
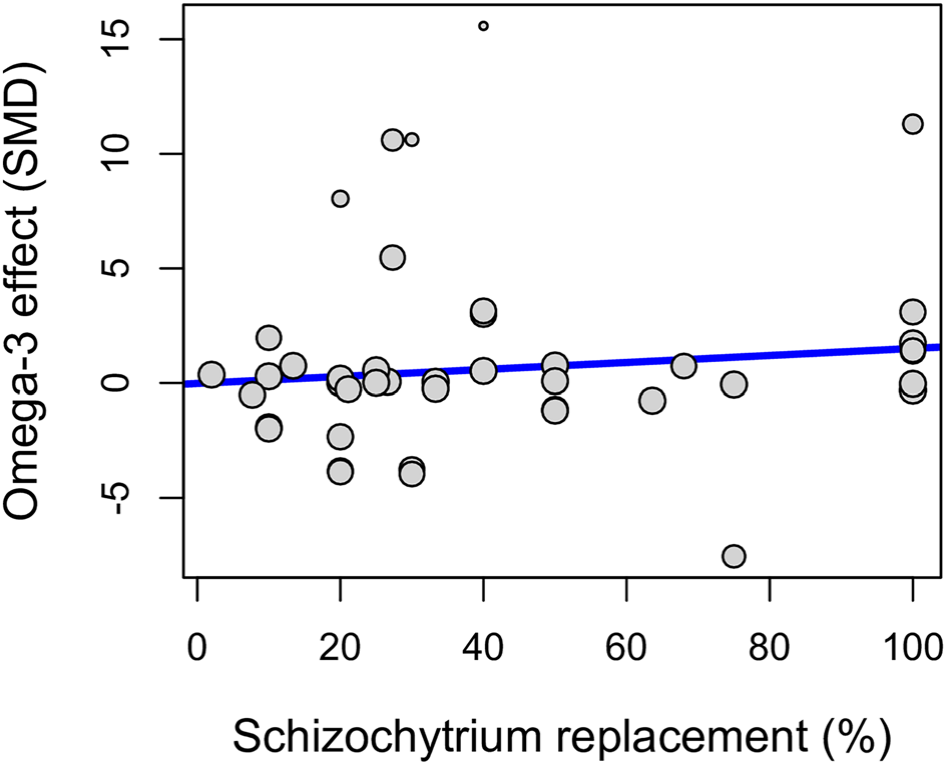
Bubble plot showing the estimated regression slope of the meta-regression on the effect of *Schizochytrium* replacement (%) on the Standardized Mean Difference in the omega-3 fillet content. The size of the points is proportional to the weight of each study.

The level of *Schizochytrium* replacement was not a significant predictor of omega-3 fillet content (*t* = 1.574, *df* = 32, *P* = 0.125; Fig. 7), but some differences were found among families. The family Terapontidae (*Scortum barcoo*, the Jade perch) showed a positive dose–effect (*t* = 2.60, *df* = 32, *P* = 0.014), although this was based on only two points from a single study^47^ and the amount of residual heterogeneity was high (*Q*_E_ = 222.53, *df* = 33, *P* < 0.001). Initial size was not a significant predictor of omega-3 fillet content (*t* = 0.102, *df* = 32, *P* = 0.919) and the best model only accounted for 20.8% of the observed heterogeneity (*F*_9,33_ = 2.252, *P* = 0.043), driven by family effects.

#### Validity of results

**Figure 8:**
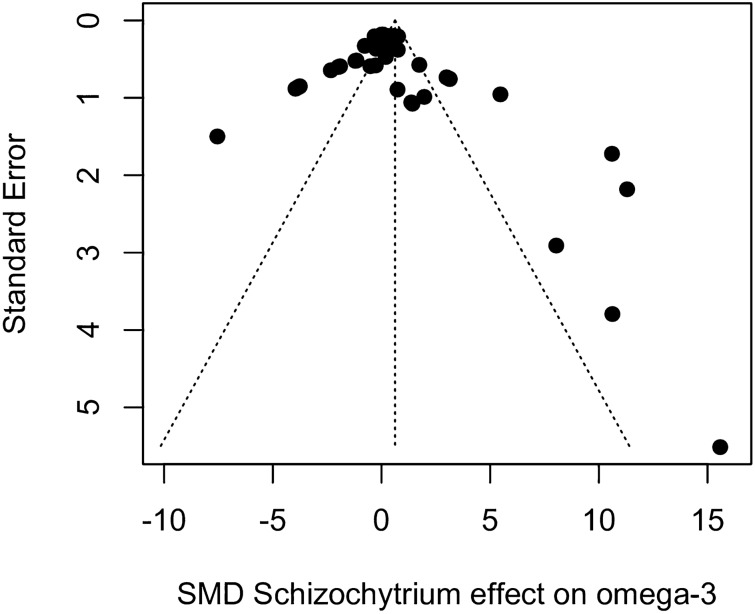
Funnel plot showing the relationship between the SMD and the standard error (inverted scale) for the effects of *Schizochytrium* replacement on omega-3 fillet content. Each point represents a treatment–control comparison and the dotted vertical line denotes the global SMD under a random effects model. An asymmetric distribution of points outside the funnel might be indicative of publication bias.

As with *Spirulina*, a funnel plot of the *Schizochytrium* SMDs against their standard errors produced an asymmetric pattern (Fig. 8) that might indicate the existence of publication bias. However, the results of an Egger’s test of funnel plot asymmetry was not significant (*t*_41_ = 0.55, *P* = 0.583; bias coefficient = 0.44, SE = 0.80), suggesting there was no conclusive evidence of publication bias.

**Figure 9:**
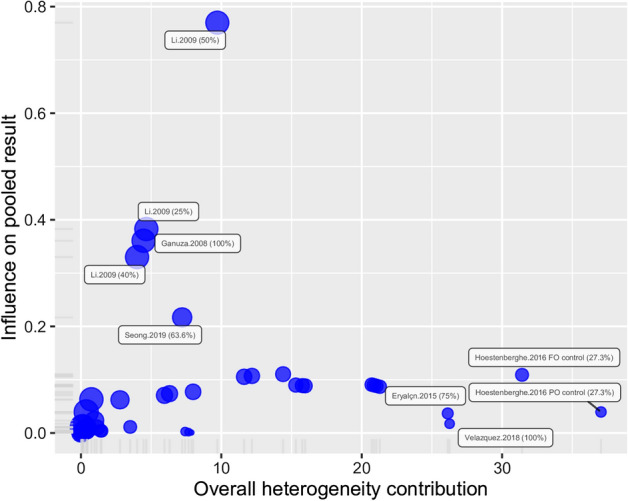
Baujat bubble plot used to identify potential outliers in the *Schizochytrium* data set, showing the contribution of each study to the overall heterogeneity and its influence under a random effects model. The size of each point is proportional to its relative weight in the meta-analysis. One trial in the upper left corner was overly influential which merited further scrutiny.

Results from the *P*-curve test indicated that the distribution of significant results was significantly right skewed (*P* binomial < 0.001, full curve *P* < 0.001; half curve *P* < 0.001), which were confirmed by the flatness test (*P* binomial > 0.999, full curve *P* > 0.999; half curve *P* > 0.999). The evidential value indicated that the observed results were robust and unlikely to have been affected by publication bias. Inspection of Baujat diagnostic plots detected one overly influential result (Fig. 9), while formal outlier analysis detected 14 extreme results. Reanalysis of the data without the overly influential point resulted in a pooled SMD of 0.63 (95% CI − 0.54; 1.80) which was not significantly different from zero (*t* = 1.09, *P* = 0.284). Removal of the 14 potential outliers resulted in a pooled SMD of 0.410 (95% CI 0.005–0.815) which was only marginally statistically significant (*t* = 2.08, *P* = 0.047).

Taken together the results indicated that although there was no convincing evidence of a positive effect of *Schizochytrium*, its inclusion did not cause a loss of omega-3 content in the fish fillet. Heterogeneity, however, was substantial even when outliers were removed (*I*^2^ = 74.4%, *Q* = 109.2, *df* = 28, *P* < 0.001).

#### Subgroup analysis

Significant differences were found in *Schizochytrium* effects with respect to fish family (*Q* = 61.70, *df* = 8, *P* < 0.001), but not with respect to habitat (*Q* = 1.59, *df* = 1, *P* = 0.208), feeding guild (*Q* = 5.96, *df* = 2, *P* = 0.051) or type of measurements (*Q* = 0.75, *df* = 1, *P* = 0.387; Table 4). Of the 9 fish families examined, two families showed a statistically significant effect of *Schizochytrium* on omega-3 content (Ictaluridare SMD = 0.530; Serranidae SMD = 1.123) but the sample size was very small, the benefits modest and the uncertainty high.

**Table 4:** Sources of heterogeneity and subgroup analysis in the *Schizochytrium* dataset according to a random effects model.

Grouping	*k*	SMD	95% CI	*Q*	*I*^*2*^
Family					
Acipenseridae	3	0.039	[− 0.037; 0.114]	0.06	0.0%
Cichlidae	8	3.214	[− 1.190; 7.618]	26.93	74.0%
Ictaluridare*	4	0.530	[0.222; 0.838]	2.70	0.0%
Moronidae	5	− 1.513	[− 4.944; 1.919]	55.02	92.3%
Salmonidae	9	0.292	[− 0.828; 1.411]	43.22	81.5%
Sciaenidae	6	0.369	[− 5.576; 6.313]	86.47	94.2%
Serranidae*	3	1.123	[0.132; 2.113]	0.32	0.0%
Sparidae	3	− 2.266	[− 12.481; 7.150]	23.49	91.5%
Terapontidae	2	7.820	[− 24.670; 40.309]	6.79	85.3%
Test for subgroup differences			*Q* = 61.70, *df* = 8, *P* < 0.001		
Habitat					
Freshwater	22	1.368	[− 0.449; 3.184]	186.80	88.8%
Marine	21	− 0.051	[− 1.537; 1.434]	179.42	88.9%
Test for subgroup differences			*Q* = 1.59, *df* = 1,* P* = 0.208		
Feeding					
Carnivores	29	− 0.305	[− 1.429; 0.819]	250.02	88.8%
Omnivores	6	2.806	[− 1.433; 7.045]	3.95	92.0%
Herbivores	8	3.214	[− 1.189; 7.618]	5.04	74.0%
Test for subgroup differences			*Q* = 5.96, *df* = 2,* P* = 0.051		
Measurement					
Individual data	33	0.132	[− 0.261; 0.525]	362.00	91.2%
Batch data	10	0.806	[− 0.743; 2.355]	14.76	39.0%
Test for subgroup differences			*Q* = 0.75, *df* = 1*, P* = 0.387		

Groups that display a positive effect of *Schizochytrium* on omega-3 content in the fish fillet are denoted by an asterisk.

*k* number of studies, *SMD* standardized mean difference compared to controls, *95% CI* 95% confidence interval around SMD, *Q* Cochran’s measure of heterogeneity.

*I*^*2*^ percentage of variability unaccounted by sampling error.

## Discussion

Microalgae offer a potential solution to the growing need for more sustainable alternatives to fishmeal and fish oils in aquafeeds, and for healthier, more nutritional substitutes to plant oils^16^, but high production costs and wide variation in the purported benefits have so far hampered a greater uptake by industry^48–50^. The potential of microalgae to serve as sustainable replacement of animal or plant based protein and oils in aquafeeds has been extensively reviewed in recent years^21,51–59^, but surprisingly there is no quantitative global assessment of their nutritional benefits. Without a statistical analysis, it is difficult to determine to what extent the nutritional benefits of microalgae can be extrapolated across species or depend on inclusion levels. For example, some authors have reported negative impacts of *Spirulina* at high inclusion levels in some species, while others have found no such constraints^51^. To address these issues, we conducted a rigorous meta-analysis on the nutritional benefits of incorporating two of the most important microalgae, *Spirulina* and *Schizochytrium,* into aquafeeds for use in fish farming, assessed the extent and sources of variation, and critically examined various potential sources of bias.

### Benefits of *Spirulina* replacement on fish growth

The results of our meta-analysis showed that partial replacement of fish meal with *Spirulina* can have a significant positive effect on fish growth, with benefits being apparent from very modest inclusion levels, 1% and less^45^. However, growth benefits were dose-dependent and higher inclusion levels of *Spirulina* resulted in better growth, 45% being the maximum *Spirulina* replacement considered. Growth was improved in 71% of the 17 species examined, but the best results occurred among the Cichlidae (tilapias), Clariidae (airbreathing catfishes), and Mugilidae (mullets), species which are all herbivorous.

**Figure 10:**
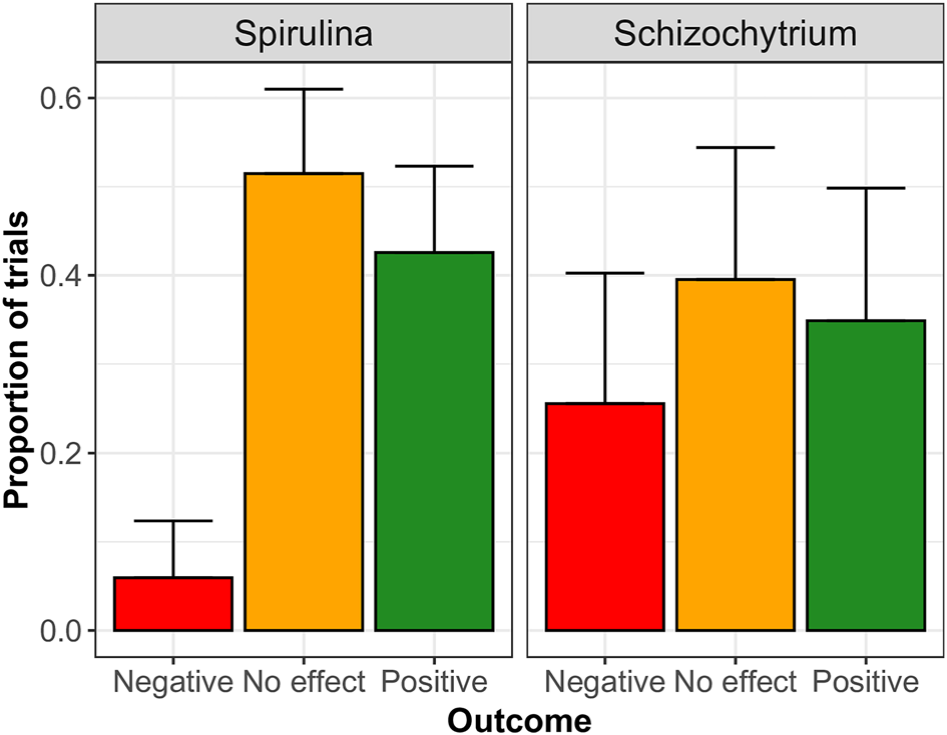
Breakdown of study outcomes (SMDs) under a random effects model for the *Schizochytrium* and *Spirulina* meta-analyses, showing the proportion of statistically significant negative effects, no effect, and positive effects along with the 95% binomial CI. The distribution of non-negative outcomes is significantly better for *Spirulina* than for *Schizochytrium* studies (*χ*^2^ = 11.197, df = 2, *P* = 0.004).

Negative results were also found, although these instances were rare. Loss of weight compared to controls following replacement with *Spirulina* was reported in 5% of studies (Fig. 10) and involved three species: Nile tilapia at 2–2.7% replacement^60,61^, mullet at 3.9% replacement^62^ and rainbow trout at 0.1–4% replacement^45,63^. In most cases (95%), however, *Spirulina* either improved growth or had no negative effect compared to controls, and replacements of up to 40–45% have been used without detrimental impacts in several species^62,64–66^.

### Benefits of *Schizochytrium* replacement on omega-3 fillet content

Ingestion of suitable sources of omega-3 PUFA is essential for proper egg development and offspring survival^67^ and *Schizochytrium* represents a sustainable and rich source of DHA for maturing fish^68^. Moreover, given the importance of the early environmental conditions for subsequent development^69,70^, the essential fatty acids provided by *Schizochytrium* and other similar thraustochytrids can have long-term beneficial effects on fish health and growth, as seen in Siberian sturgeon^71^, Nile tilapia^26,27,46,72^, red sea bream^73^, channel catfish^74^, and jade perch^47^.

We did not find a positive global increase in omega-3 in the fish fillet compared to controls, but the mean SMD was not statistically different from zero, indicating that replacement of fish or plant oil with *Schizochytrium* oil is possible without a significant loss of omega-3 content. Indeed, positive or neutral (i.e., zero-effect) results were reported in 74% of the trials (Fig. 10). The 26% of cases where the omega-3 content of the fish fillet deteriorated after *Schizochytrium* inclusion referred to studies involving five species: red drum^75^, hybrid striped bass^76^, Atlantic salmon^77^, red seabream^78^, and gilt-head bream^79^. The absence of a dose effect means that 100% substitution of animal or plant oils with *Schizochytrium* oil is possible and should not decrease the nutritional value of the fish fillet, as demonstrated for Nile tilapia^26^, although variability is very high and the prediction interval wide, which introduces considerable uncertainty on the expected results.

### Heterogeneity between studies and sources of variation

We found substantial heterogeneity in the results of fish feeding studies using *Spirulina* (*I*^2^ = 96%) and *Schizochytrium* (*I*^2^ = 89%)*.* The relative frequency of different outcomes (positive, neutral, and negative results) differed significantly between *Spirulina* and *Schizochytrium* studies *(χ2* = 11.197, *df* = 2, *P* = 0.004; Fig. 10). Non-negative results (i.e. positive plus neutral) were more common for *Spirulina* effects on growth (94%) than for *Schizochytrium* effects on omega-3 fillet content (74%), confirming the results of the two meta-analyses, which yielded a significant non-zero global effect for *Spirulina* (95% CI SMD = 0.71–1.70) but included zero in the case of *Schizochytrium* (95% CI SMD = − 0.51–1.76). Highly variable outcomes are common in microalgal studies. For example, Ahmad et al.^80^ reported 36% significant improvements in 11 studies that examined changes in growth or fillet quality following inclusion of *Chlorella vulgaris* in aquafeeds, 36% with no discernible benefit, and 27% negative effects, which were apparently exacerbated at high inclusion levels.

High heterogeneity in meta-analysis is problematic because it makes it difficult to generalize across contexts^35,81^. Heterogeneity can be caused by clinical (or structural) differences between subjects and how they respond to treatments, but also by methodological differences in study design, and by statistical variation in intervention effects^82,83^. We dealt with high heterogeneity by performing meta-regression and by conducting subgroup analysis^84^. We found that family effects were the main source of heterogeneity, but this only explained a small part of the observed variation (~ 24–27%). Most of the variation could not be explained by differences in the way different fish families responded to microalgae replacement, or by variation in microalgae inclusion levels, differences in fish size, habitat, feeding guild or the way the data were recorded.

It is likely that other, unaccounted, biotic and abiotic sources of variation contributed to the high observed level of heterogeneity^85^. For example, fish growth can vary enormously depending on sex and stocking density^86^, water temperature^87^, photoperiod^88^, light intensity^89^, tank size^90^, tank colour^89^, social status^91^, trial duration, seasonality and feeding rates^92^. These are likely to differ between studies but are seldom reported. Likewise, substantial variation has also been reported in the fatty acid composition of fish fed identical diets under communal rearing conditions^93^, suggesting that individual differences in deposition of omega-3 can be substantial. The nutritional value of micro-algae also differs between strains and producers^94^, depending on culture conditions^95^, geographic location^96^ and post-harvest treatment^97–99^, adding additional sources of unaccounted variation.

### Publication bias

We found no clear evidence of systematic publication bias. Plotting effect sizes against standard error of the estimates resulted in asymmetric funnel plots for both *Spirulina* and *Schizochytrium* which can be indicative of publication bias^100^. However, asymmetry could not be confirmed by the more explicit Egger’s tests^40^ in the case of *Schizochytrium* and the results of *p*-curve analysis^42^ indicated that there was sufficient evidential value for both micro-algae, suggesting there was an underlying true effect. Publication bias could have been masked by high study heterogeneity which may have diminished the power of the p-curve method^101^, but our sensitivity analysis indicates that the pooled effect sizes calculated for *Spirulina* and *Schizochytrium* were robust to the exclusion of outliers and overly influential points.

### Wider benefits of using microalgae in aquafeeds

There are over 40 different species of micro-algae used in fish farming, but these are mostly used to feed rotifers and copepods to wean fish larvae, or are administered live directly to fish reared in ‘green waters’^30,102^. Only ~ 19 microalgae are used as part of formulated aquafeeds^16,103^, the production being dominated by freshwater species such as *Spirulina*, which is the dominant species with 41% of the global market due its ease of culture, nutrient profile and high yield^104^.

Although live microalgae are a staple feed in many fish hatcheries^105^, ingestion rates are difficult to control in ‘green waters’ and their use is typically restricted to larval stages. In contrast microalgae-based aquafeeds can be used at all stages of fish development, offering superior control over feeding, necessary for precision aquaculture^106^. Also, unlike plant-based aquafeeds that are difficult to be accepted by carnivorous species^107^, microalgae incorporated into aquafeeds can be used to feed both carnivorous and herbivorous species^59^. Many microalgae have rigid cell walls which results in low digestibility^108^, but new technical solutions are being developed to overcome this challenge^59,109,110^.

Not all species are as rich in omega-3 PUFA as *Schizochytrium*^26^, or have the high protein content of *Spirulina* (~ 63–65%) to replace fish meal^98^, but combining different microalgae can overcome this limitation. For example, *Schizochytrium* represents a good source of DHA for maturing fish, but is poor in EPA^68^, but by combining it with oil from *Nannochloropsis* which is rich in EPA^111^ an appropriate balance of omega-3 fatty acids can be ensured, necessary for the production of high quality gametes^112^. Likewise, while *Schizochytrium* oil possess a nutritional profile comparable to fish oil^26,27,96^, *Spirulina* lacks essential amino-acids compared to fish meal, which can potentially reduce growth at high inclusion levels for some species^21,113^. Thus, different combinations of microalgae may be required to meet the nutritional needs of different fish species^114^. Yet, few studies have compared the benefits of combining different proportions of microalgae and this is an area where more research is clearly needed.

One advantage of microalgae over plant-based aquafeeds is that their benefits are not limited to enhanced growth or nutritional value, but can also extend to fish health^16,115^. Microalgae are increasingly being considered for their therapeutic properties, in addition to their nutritional aspects^116^. For example, *Spirulina* and *Chlorella* can boost the immune system of fish^80,117^, and *Spirulina* may also have anti-viral properties^118^. Incorporation of *Spirulina* in the fish diet was reported to enhance hepatic antioxidant function and disease resistance in coral trout, *Plectropomus leopardus*^119^, great sturgeon *Huso huso*^120^, Nile tilapia^60,121,122^, African catfish^123^, mullet^124^, as well as in several cyprinids^24,125^ and salmonids ^45,126^. Inclusion of *Spirulina* at 8–10% was also found to increase fecundity in three-spot gourami^127^.

### Maximizing the value of feeding studies using microalgae

Microalgae can provide substantial benefits to aquaculture nutrition but only if results can be replicated and can be used by the aquafeed industry^7,8^. In common with other meta-analysis in aquaculture^128^, we found it difficult to extract the necessary information from fish feeding trials to ascertain effect sizes. A surprisingly large number of studies did not provide enough information to replicate the work, or to ascertain the experimental validity of the results. Of 1474 studies we screened, only 3% were eligible for analysis. Few studies adhered to accepted guidelines for reporting fish feeding trials, failing to report mean effects, sample sizes and measures of variability^129^, or ethical considerations^130^.

In the studies reviewed, 14% of trials involved batch measurements in the case of *Spirulina* and 23% in the case of *Schizochytrium,* and this may have also introduced some biases. Batch measurements are not recommended as they can mask important sources of variation, reduce sample size (and thus statistical power) and may result in inflated effect sizes, which can be misleading. It might be beneficial for future meta-analysis to weigh studies by some measure of reliability^41,81^.

The unit of replication should also take into account the nested nature of the data and the statistical power to detect differences, particularly in growth studies^131,132^. For example, there is little benefit in using triplicate tanks if tank effects are ignored and data are pooled. Fish can now be individually marked since a young age^133^, which is essential for precision fish farming^106^, and tank effects can be accounted for using linear mixed effects models^131^.

All results we reviewed were based on feeding trials typically carried out in comparatively small tanks or enclosures under relatively low densities, which are unlikely to be representative of commercial conditions. Given the high heterogeneity found in effect sizes, there is some uncertainty about the wider applicability of the reported results. There is clearly a need to examine the performance of algae-enriched aquafeeds under commercially relevant conditions that extend over longer time periods than the average 60-day feeding trial to ascertain the validity and potential limitations of upscaling^134^.

### Outlook and conclusions

Although our meta-analyses examined the nutritional benefits of only two species of microalgae, these represent the main ones, and were the only ones with enough quantitative data on nutritional benefits. The results indicate that inclusion of *Spirulina* in the fish diet improves specific growth rate overall, while replacement of fish or plant oil with *Schizochytrium* oil is possible without loss of omega-3 content in the fish fillet in the majority of studies and species examined. However, the results were very heterogenous and the nutritional benefits depended on fish species, and in the case of *Spirulina* also on inclusion levels.

The Aquaculture industry will be worth $50.6 billion by 2026^135^, the main cost of which will continue to be the cost of aquafeeds^136^. The use of microalgae in aquafeeds is still more expensive than using fishmeal, fish oils or plant crops^48^ but the price of fish meal has increased more than 200% over the last two decades^137^. As microalgae production becomes cheaper and more efficient^20^, microalgal-based aquafeeds will become more competitive^138^. Production of *Spirulina* is expected to be worth $4.6 billion by 2027^139^, mostly driven by the nutraceutical, food and beverage segment, but also by aquaculture^140^. To speed the transition towards more sustainable, zero-catch aquafeeds, we recommend that feeding trials using microalgae are conducted under commercially relevant conditions and that the raw data and full rearing details are fully reported to facilitate comparative analyses.

## Data Availability

The datasets analysed in this study are all in the public domain and available from the sources listed in Tables 1 and 3. The data used in the meta-analysis is available from the corresponding author on reasonable request.
